# The COVID-19 pandemic should not derail global vector control efforts

**DOI:** 10.1371/journal.pntd.0008606

**Published:** 2020-08-31

**Authors:** Frederik Seelig, Haroldo Bezerra, Mary Cameron, Jeffrey Hii, Alexandra Hiscox, Seth Irish, Robert T. Jones, Trudie Lang, Steven W. Lindsay, Rachel Lowe, Tanaka Manikidza Nyoni, Grace M. Power, Juliana Quintero, Anna M. Stewart-Ibarra, Lucy S. Tusting, Scott Tytheridge, James G. Logan

**Affiliations:** 1 The Global Vector Hub, Department for Disease Control, London School of Hygiene & Tropical Medicine, London, United Kingdom; 2 Department of Communicable Diseases and Environmental Determinants of Health, Neglected, Tropical and Vector-Borne Diseases, Pan-American Health Organization/World Health Organization, Washington, DC, United States of America; 3 College of Public Health, Medical and Veterinary Sciences, James Cook University of North Queensland, Townsville, Australia; 4 ARCTEC, London School of Hygiene & Tropical Medicine, London, United Kingdom; 5 U.S. President’s Malaria Initiative and Entomology Branch, Division of Parasitic Diseases and Malaria, Center for Global Health, Centers for Disease Control and Prevention, Atlanta, Georgia, United States of America; 6 The Global Health Network, Nuffield Department of Medicine, University of Oxford, Oxford, United Kingdom; 7 Department of Biosciences, Durham University, Durham, United Kingdom; 8 Centre for Mathematical Modelling of Infectious Diseases, London School of Hygiene & Tropical Medicine, London, United Kingdom; 9 Centre on Climate Change and Planetary Health, London School of Hygiene & Tropical Medicine, London, United Kingdom; 10 Barcelona Institute for Global Health (ISGlobal), Barcelona, Spain; 11 Department of Infectious Disease Epidemiology, London School of Hygiene & Tropical Medicine, London, United Kingdom; 12 Department of Population Health, Fundación Santa Fe de Bogotá, Bogotá, Colombia; 13 Inter-American Institute for Global Change Research (IAI), Montevideo, Uruguay; 14 Department of Medicine, State University of New York Upstate Medical University, Syracuse, New York, United States of America; Faculty of Science, Mahidol University, THAILAND

The COVID-19 pandemic is placing immense pressure on health systems worldwide. This is particularly apparent in resource-poor settings with limited capacity to treat and contain new disease outbreaks.

The World Health Organization (WHO) has emphasised the crucial need to sustain efforts to prevent, detect, and treat malaria during this pandemic [1]. However, a similar approach should also be adopted for the control of arboviral diseases of global importance, including dengue, Zika, chikungunya, and yellow fever, as recommended by the Pan-American Health Organization (PAHO) in their interim guidance on control of *Aedes aegypti* mosquitos during the COVID-19 pandemic [2].

For example, an unprecedented dengue epidemic continues to affect Latin America and the Caribbean, with a surge of cases experienced in the first half of 2020 [3]. Even before the COVID-19 pandemic began, many countries were struggling to control dengue. The public health interventions initiated to halt the COVID-19 spread have already severely affected routine vector surveillance and control activities (such as regular household surveys) [4,5]. The combined impact of both COVID-19 and epidemics of dengue or other vector-borne diseases (VBDs) could have potentially devastating consequences [6].

In view of these combined challenges, we reiterate our solidarity with the global partners who are dealing with the COVID-19 pandemic, while strongly urging them to consider these recommendations for the control of VBDs:

- Continue the implementation of the WHO’s global vector control response 2017–2030 (GVCR) strategy and regional policies for vector control [7,8], with respect to inter- and intrasectoral collaboration, engagement and mobilisation of communities, and scaling up of vector control if required, according to the implementation plan of vector control activities, while adapting activities as necessary to prevent further spread of COVID-19, in particular vector surveillance, which may need to be scaled down [9,10].

And, more specifically with respect to VBD control, we present a list of recommended actions grouped into three categories:

1. Maintain vector control operations in the context of COVID-19 (adapting activities to improve worker safety and to prevent further spread):
  - Continue and enhance protection of vulnerable populations against VBDs, e.g., through providing topical repellents and distributing bed nets, and deliver vector control safely while practising physical distancing.
  - Ensure the safety of community workers and volunteers through training on safe methods to conduct their work in the face of COVID-19 and provide personal protective equipment (see section 2.5 in [2] for further details).
  - Perform intensive risk assessment and contingency planning for continuity of vector control by dedicated health personnel with appropriate training. This may involve moving from community-based control to household-, clinic-, hospital-, or school-level control measures.
2. Broad programmatic considerations for vector control activities during outbreaks of other aetiologies:
  - Encourage global funding bodies and dedicated donors to provide long-term funding for capacity building in vector surveillance and control, including training and retention of local staff, and for contingencies during any anticipated lockdown. It is imperative not to reduce existing funding on vector control programmes or to lose sight of the value of vector control, which would offset any previous gains.
  - Improve the validity, availability, and proper use of rapid testing, both for COVID-19 and VBDs, to prevent misdiagnosis and to improve case management for potential coinfections and comorbidities [11].
  - Identify gaps in practices and knowledge related to vector-borne disease control and encourage local and national stakeholders to address these deficiencies.
  - Improve preparedness for outbreaks (vector-borne or not) by devising a comprehensive preparedness plan that will identify all activities and materials required for each stage (from no risk to epidemic) and by providing strategic reserves of pharmaceuticals, personal protective equipment, public health insecticides, and vector control equipment.
  - Despite significant progress to date, many research activities are currently on hold. The search for new and effective tools for both malaria and dengue control must continue, including the use of artificial intelligence and novel technological solutions, such as drones, to assist in vector surveillance and control.
  - Integrate modelling to predict the combined impact of VBDs and COVID-19.
3. COVID-19 disease monitoring and control innovations that could be applied to vector control:
  - One of the encouraging aspects of the response to the COVID-19 pandemic has been the rapid and open global dissemination of data, such as virus sequence data. This has allowed development of the first vaccine candidates in record time. We strongly recommend a similar effort to promote relevant data sharing on vectors, arboviruses, and other VBDs to support public health interventions. The Global Vector Hub (GVH) online platform was designed to facilitate easy access and exchange of information, data, and resources among relevant stakeholders, and an early version was launched in June 2020 to support global vector control efforts in the context of the COVID-19 pandemic.
  - Provide accurate and up-to-date information and data from trusted sources to fight the spread of misinformation.
  - Support studies that map and evaluate the impact of distancing and isolation measures on vector control interventions and VBDs, especially in large urban centres.

It is expected that the most vulnerable and poorest populations will suffer most from COVID-19, due to lack of access to appropriate care and the impacts of isolation measures on fragile livelihoods. It is vital that the COVID-19 response does not increase VBD threats in these communities by derailing global vector control efforts.
